# Oxidative Stress in Obesity: A Critical Component in Human Diseases

**DOI:** 10.3390/ijms16010378

**Published:** 2014-12-26

**Authors:** Lucia Marseglia, Sara Manti, Gabriella D’Angelo, Antonio Nicotera, Eleonora Parisi, Gabriella Di Rosa, Eloisa Gitto, Teresa Arrigo

**Affiliations:** 1 Neonatal and Pediatric Intensive Care Unit, Department of Pediatrics, University of Messina, Via Consolare Valeria 1, 98125 Messina, Italy; E-Mails: gabridangelo@alice.it (G.D.); egitto@unime.it (E.G.); 2 Unit of Paediatric Genetics and Immunology, Department of Paediatrics, University of Messina, Via Consolare Valeria 1, 98125 Messina, Italy; E-Mails: saramanti@hotmail.it (S.M.); tarrigo@unime.it (T.A.); 3 Unit of Child Neurology and Psychiatry, Department of Pediatrics, University of Messina, Via Consolare Valeria 1, 98125 Messina, Italy; E-Mails: antonionicotera@ymail.com (A.N.); leluccia83@hotmail.it (E.P.); gdirosa@unime.it (G.D.R.)

**Keywords:** obesity, oxidative stress, adipocytokines, human diseases, adipose tissue

## Abstract

Obesity, a social problem worldwide, is characterized by an increase in body weight that results in excessive fat accumulation. Obesity is a major cause of morbidity and mortality and leads to several diseases, including metabolic syndrome, diabetes mellitus, cardiovascular, fatty liver diseases, and cancer. Growing evidence allows us to understand the critical role of adipose tissue in controlling the physic-pathological mechanisms of obesity and related comorbidities. Recently, adipose tissue, especially in the visceral compartment, has been considered not only as a simple energy depository tissue, but also as an active endocrine organ releasing a variety of biologically active molecules known as adipocytokines or adipokines. Based on the complex interplay between adipokines, obesity is also characterized by chronic low grade inflammation with permanently increased oxidative stress (OS). Over-expression of oxidative stress damages cellular structures together with under-production of anti-oxidant mechanisms, leading to the development of obesity-related complications. The aim of this review is to summarize what is known in the relationship between OS in obesity and obesity-related diseases.

## 1. Oxidative Stress and Obesity

Obesity, characterized by an increase in body weight that results in excessive fat accumulation, represents a social problem worldwide [1] and has been recognized as a major underlying factor in the pathogenesis of several diseases [2]. Unfortunately, obesity also involves a growing number of children in developed countries. Moreover, it has been reported that children and adolescents who are obese are likely to be obese as adults [3] and are therefore more at risk for adult health problems [4]; One study has assessed that children who became obese as early as age 2 were more likely to be obese as adults [3]. Recently, it has also been found that obesity is associated with low-grade chronic systemic inflammation in adipose tissue. This condition is influenced by the activation of the innate immune system in adipose tissue that promotes pro-inflammatory status and oxidative stress (OS), triggering a systemic acute-phase response. Several chronic diseases are also the result of obesity (e.g., metabolic syndrome, diabetes mellitus, liver and cardiovascular diseases, and cancer) and associated with OS [2]. Therefore, it has been hypothesized that inflammation of adipose tissue in obese patients plays a critical role in the pathogenesis of obesity-related complications [5].

**Figure 1:**
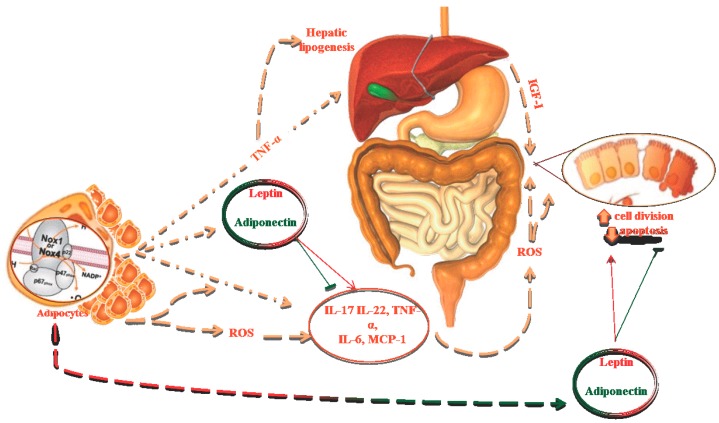
Underlying pathophysiological mechanisms of cancer susceptibility in obese patients.

Adipose tissue is an endocrine and storage organ required for energy homeostasis. This tissue, primarily composed of adipocytes, also contains other cells (e.g., fibroblasts, fibroblastic pre-adipocytes, endothelial and immune cells) [6], secreting hormones and cytokines (adipokines or adipocytokines) which exercise endocrine, paracrine, and autocrine action on the whole body. In physiological and, even more, in pathological conditions, adipokines also induce the production of reactive oxygen species (ROS), generating OS and, in turn, a major, irregular production of other adipokines [7]. Several mechanisms are involved in generating OS in obesity (Figure 1). OS and pro-inflammatory processes are strongly related [8,9]. Upon activation, many immune cells generate free radicals (FR) and, in the same way, the synthesis of ROS promotes an inflammatory status.

Firstly, the presence of excessive adipose tissue has been identified as a source of pro-inflammatory cytokines including tumour necrosis factor-alpha (TNF-α), interleukin (IL)-1β, and IL-6 [10].

TNF-α is a critical cytokine that influences the inflammatory response, the immune system, adipose cell apoptosis, as well as lipid metabolism, increasing hepatic lipogenesis, insulin signalling and inducing OS. ROS production can also be induced by TNF-α through binding of specific receptors and promoting NF-κB signalling [11]. Serum TNF-α levels are increased in obesity and decreased with weight loss. TNF-α favours the systemic acute-phase response, via the release of IL-6, another pro-inflammatory molecule, and via the reduction of systemic anti-inflammatory cytokines, like adiponectin. TNF-α also increases the interaction of electrons with oxygen to generate superoxide anions [12].

IL-1β, a pyrogenic cytokine, is mainly released by monocytes in response to tissue damage, infection, or immunologic challenge. Recently, it has been assessed that IL-1β is an instigator of the pro-inflammatory response in obesity via production of additional pro-inflammatory cytokines, such as IL-6 [13].

IL-6, secreted by a wide variety of cells (e.g., adipocytes, endothelial cells, β-pancreatic cells, macrophages, and monocytes), regulates energy homeostasis and inflammation, influencing the transition from acute to chronic inflammatory disease, such as obesity and insulin resistance [14], by promoting the synthesis of pro-inflammatory cytokines and by negatively regulating inflammatory targets. In humans, higher serum IL-6 levels have been associated with elevated likelihood of impaired glucose tolerance, diabetes mellitus, high blood pressure, and especially obesity. Visceral adipose tissue, secreting other molecules that stimulate further IL-6 expression, releases approximately two to three times more IL-6 than subcutaneous tissue, [15]. IL-6 can also suppress lipoprotein lipase activity, and control appetite and energy intake at a hypothalamic level [16].

Finally, accumulated adipose tissue induces the synthesis of pro-inflammatory cytokines, including TNF-α, IL-1, and IL-6, which promote increased generation of ROS and nitrogen by macrophages and monocytes; therefore, a rise in concentration could be responsible for increased OS [10]. ROS induce the further release of pro-inflammatory cytokines and expression of adhesion molecules and growth factors (e.g., connective tissue growth factor, insulin-like growth factor-1 (IGF-I), platelet-derived growth factor, and vascular cell adhesion molecule-1) [17] through redox-sensitive transcription factors, particularly NF-κB and the NADPH oxidase pathway (NOX) [18]. NOX, especially NOX4 [19], is a membrane-bound enzyme complex that transfers electrons from NADPH to oxygen and represents a major source of ROS synthesis in adipocytes. Generated O_2_ radicals are further converted into hydrogen peroxide (H_2_O_2_), longer-lived membrane-permeable ROS. H_2_O_2_ also stimulates IL-4 and IL-6 gene expression and cytokine secretion by an apurinic/apyrimidinic-endonuclease/redox-factor-1- (APE/Ref-1-) dependent pathway [20]. Confirming these data, experimental models have reported that the silencing of oxidant sources (NOX4) inhibits palmitate- and glucose-stimulated ROS generation, underlying the importance of NADPH oxidases as a non-mitochondrial source of ROS in adipocytes [21]. Nevertheless, a strong cross talk between NAPDH and mitochondria also exists. Mitochondria constitute a target for ROS produced by NOX but also a significant source of ROS, which in turn can further stimulate NADPH oxidases [22]. It has also been reported that mitochondria-targeted antioxidants inhibit ROS production by mitochondria, reducing NOX activity [23].

Susceptibility to oxidative damage is even greater in obese subjects because of depleted antioxidant sources, including superoxide dismutase (SOD), glutathione peroxidase (GPx), and catalase (CAT), vitamin A, vitamin E, vitamin C, and β-carotene [24]. Compared to normal weight patients, the activity of SOD in obese individuals is significantly lower [25]. Moreover, it has been demonstrated that anti-oxidant supplementation could reduce OS and ROS, decrease the risk of complications related to obesity, and restore expression of adipokines [26].

Secondly, although during the period of active growth of organisms increased free fatty acids (FFA) levels are physiologically observed, excessive fat accumulation in obese patients leads to a pathological increase of serum FFA levels which, in turn, impairs glucose metabolism [27], favour hepatic, muscular, and adipose accumulation of energy substrates (fats and glucose) [28], and promotes higher mitochondrial and peroxisomal oxidation. This status leads to major synthesis of free radicals (FR), OS, mitochondrial DNA injury, depletion of adenosine triphosphate (ATP) [29], and, finally, lipotoxicity, involving various negative effects of fatty acids on cellular structures [30]. Cellular damage leads to high production of cytokines such as TNF-α, which generates further ROS in tissues and increases the lipid peroxidation rate [31].

Thirdly, it should be noted that adipose tissue is a source of bioactive adipokines, including leptin, adiponectin, visfatin, resistin, apelin, and plasminogen activator inhibitor type 1 (PAI-1), implicated in the homeostasis of physiological and pathological processes involving OS.

Leptin is a hormone mainly secreted by adipocytes in direct proportion to the mass of adipose-tissue and to triglyceride (TG) storage-adipose. It is primarily known for its anorexigenic action, as it circulates in plasma bound to proteins and, entering by diffusion into the central nervous system (CNS), causes satiety. Nevertheless, obesity is associated with increased leptin levels and it has been postulated that the apparent decrease in anorexigenic effects and weight loss are the result of a mechanism of resistance to it [10]. It is less well known that leptin promotes OS, increasing phagocytic activity of macrophages, inducing pro-inflammatory cytokine synthesis (TNF-α, IL-6, IL-2), and interferon-gamma (IFN-γ), exerting its effect on several cells (e.g., T-cells, monocytes, neutrophils, and endothelial cells) [32], and also increasing levels of markers of endothelial cell dysfunction and activation [33]. Authors have hypothesized that pro-inflammatory effects of leptin are related to structural and functional similarities with the IL-6 family of cytokines [34]. Studies have also reported that increased levels of C-reactive protein (CRP) are detected when leptin is administered, further confirming inflammatory effects. According to these data, during weight loss, circulating leptin levels and obesity-associated inflammatory markers are reduced [20].

In contrast to leptin, adiponectin, secreted by differentiated adipocytes [35], shows high anti-inflammatory and anti-atherogenic powers as it inhibits adhesion of monocytes to endothelial cells, transformation of macrophages into foam cells, and endothelial cell activation; it also decreases TNF-α and CRP levels, and increases nitric oxide (NO) production in endothelial cells [36]. Additionally, adiponectin inhibits ROS release mediated by low-density lipoprotein (LDL). Adiponectin deficiency results in NO reduction and leukocyte adhesion, causing chronic vascular inflammation [36]. It was observed that TNF-α and IL-6 are potent inhibitors of adiponectin synthesis as well as of other adipocytokines, including visfatin [35]. Finally, exposition of adipocytes to high ROS levels suppresses adiponectin expression and secretion [26]. Accordingly, human serum adiponectin levels have been inversely correlated with systemic OS [37].

Visfatin, a relatively recently discovered adipokine, is mostly expressed in human visceral fat [38,39] although it is synthesized by bone marrow, liver, lungs, skeletal muscle, brain, heart, pancreas, and peripheral blood lymphocytes. Plasma visfatin levels have been positively correlated with body fat mass and concentration decreases when weight loss occurs [40]. It is a pleiotropic molecule showing pro-oxidant and pro-inflammatory effects. In experimental research, Moschen and colleagues demonstrated that visfatin, whose serum levels are higher in patients with inflammatory disease, including obesity, than in healthy subjects [41], induced human leukocytes and pro- and anti-inflammatory cytokine production (IL-1b, IL-1Ra, IL-6, IL-8, IL-10, and TNF-α) [41]. Moreover, visfatin generates ROS comprising both superoxide and H_2_O_2_ and producing OS. However, visfatin-induced OS occurs independent of activation of the mitogen-activated protein kinases (MAPKs). In contrast, phosphorylation of the NF-κB pathway is associated with visfatin-mediated generation of ROS, and blockade of this pathway via selective IkB kinase (IKK) inhibition leads to a partial reduction in OS [42].

Resistin, expressed in lower levels in adipocytes but at relatively higher levels in circulating blood monocytes, was originally described as an adipokine involved in appetite regulation, energy balance, and insulin resistance. Thereafter, widespread research on the relationship between resistin and obesity highlighted its role in OS-related cardiovascular disease. Resistin promotes endothelial cell activation and upregulates several adhesion molecules and pro-inflammatory vascular cytokines [43]. An increase in resistin concentration significantly decreases endothelial nitric oxide synthase (NOS) expression and NO production through OS in cultured human coronary artery endothelial cells [44], suggesting that the effects of resistin can be mediated by OS.

Apelin is another short peptide released by adipocytes in proportion to the amount of fat present, and possesses anorectic properties accompanied by increased body temperature and locomotor activity, as well as inhibiting the secretion of glucose-dependent insulin. Apelin also causes NO-mediated, endothelium-dependent vasodilation and endothelium-independent vasoconstriction on smooth muscle cells [10]. Although serum apelin levels are increased in obesity associated with insulin resistance and hyperinsulinemia [38], its regulatory role on OS in adipocytes remains unknown. Recently, authors have provided evidence that apelin, through its interaction with specific apelin receptors (APJ), suppresses production and release of ROS in adipose tissue. This is further supported by observations that apelin promotes the synthesis of anti-oxidant enzymes via MAPK kinase/ERK and AMP-Activated Protein Kinase (AMPK) pathways, and suppresses the expression of pro-oxidant enzymes via the AMPK pathway. Moreover, apelin can also relieve OS-induced dysregulations of the expression of anti- and pro-oxidant enzymes, mitochondrial biogenesis and function, as well as release of pro- and anti-inflammatory adipocytokines [45]. Confirming these data, studies have reported that apelin and its structural analog involve reduction of short-lived ROS generation and improvement of the antioxidant state in OS-related conditions [46].

Although plasma levels of PAI-1 are regulated on a genetic basis, human adipose tissue, especially visceral fat, has also attracted considerable attention as a source of a predominant inhibitor of the fibrinolytic system. In addition to contributing to thrombus formation and the development of cardiovascular disease, PAI-1 can play an important role in the regulation of adipose tissue [47], increasing the blood flow of fatty acids and the risk of insulin resistance. These effects are mediated by release of pro-inflammatory cytokines [1] as well as NF-κB activation, inducing OS [48]. PAI-1, favouring elevated TGF-β1 levels, is causatively linked to the activation of inflammatory signalling pathways and OS [49]. Moreover, although the additive activation of *PAI-1* gene transcription by OS could explain the increase in circulating PAI-1, [50] it is not well understood how OS mediates the production of PAI-1. Accumulating evidence highlights the notion that hypoxia may exist in fat depots as tissue mass increases [51]. Thus, adipocyte hypertrophy might lead to the presence of local hypoxic areas that, through hypoxia-inducible factor (HIF)-1α, increase expression of several pro-inflammatory cytokines (TNF-α, IL-6) and ROS which lead to a higher expression of PAI-1 in adipocytes [52].

In conclusion, dysfunction of adipose tissue may induce systemic OS and, in turn, OS is associated with an irregular production of adipokines, which contributes to the development of pathological systemic consequences. Moreover, the sensitivity of biomarkers of oxidative damage are higher in obese individuals and correlate directly with body mass index (BMI) and the percentage of body fat, LDL oxidation, and triglyceride (TG) levels [53]; in contrast, antioxidant defense markers are lower according to amount of body fat and central obesity [54].

## 2. Oxidative Stress, Obesity and Metabolic Syndrome

According to the International Diabetes Federation, metabolic syndrome (MS) is characterized as the presence of three or more of the following features: Obesity, hyperglycemia, hypertension, low high-density lipoprotein (HDL) cholesterol levels, and/or hypertriglyceridemia [55]. Although the mechanistic role of MS pathophysiology has not been fully elucidated, obesity is considered as a pivotal component in MS [56]. It has been hypothesized that dysregulated production of adipocytokines (PAI-1, leptin, resistin, visfatin, adiponectin) and cytokines (TNF-α and IL-6) from accumulated fat participates in the pathogenesis of obesity-associated MS. Increased plasma PAI-1 and TNF-α levels contribute to the development of thrombosis and insulin resistance [32], respectively. In MS patients, several reports have demonstrated increased IL-6 levels related to BMI and insulin resistance [57]. In particular, IL-6 seems to induce insulin resistance impairing hepatic signalling and affecting the phosphorylation of insulin receptor substrate 1 (IRS-1), glucose transporter 4 (GLUT–4) [58], and other specific transcription factors [59]. The role of leptin in MS pathophysiology has also been demonstrated; it affects insulin sensitivity, and induces insulin resistance and lipid accumulation [60]. Similar to leptin effects, resistin seems to mediate insulin resistance [61]. Visfatin might also play a critical role in MS pathophysiology; serum levels, correlated to lipid metabolism and inflammatory response, contribute to decreased function of pancreatic β-cells [62]. Conversely, a protective role of adiponectin against MS has recently been reported. This molecule inhibits activity and release of IL-6 and TNF-α, and increases IL-10 and IL-1Ra production in adipocytes and macrophages [63]. Apelin also reduces MS risk and, in obesity, increased adipose and systemic levels of apelin have been detected [63].

Although dysregulated production of “offensive” adipocytokines in obese patients is strongly associated with MS [64], recent studies have shown that OS is also critically involved in the pathogenesis of MS. OS is known to impair both insulin secretion by pancreatic β-cells [65] and glucose transport in muscle [66] and adipose tissue [67]. Increased OS in vascular walls is involved in the pathogenesis of atherosclerosis, hypertension, and hepatic steatosis [64]. OS, locally produced in each of the above tissues, induces damage to cell structures, including membranes, proteins, and DNA, and, for these reasons, OS would appear to be involved in the pathogenesis of each disease leading to MS [68]. Firstly, visceral fat accumulation induces an increase in systemic lipid peroxidation and damage through excess FFA and cytokines like TNF-α, which then triggers systemic oxidative damage [69]. Secondly, patients with MS showed lower anti-oxidant activities [68]. With regard to hypertension, antioxidant and oxidant imbalance is a well-known physiological regulator of arterial pressure, and recent studies noted that OS causes endothelial dysfunction, leading to increased blood pressure and coronary artery disease [68]. Regarding dyslipidemia, many *in vitro* and *in vivo* studies have reported higher ROS release, and lower SOD and eNOS synthesis in dyslipidemia [68].

Considering the strong associations between OS, markers related to OS, antioxidant status and MS [70] some researchers hypothesized that OS is an early event and/or a candidate for a pivotal role in the pathology of MS [68]. Moreover, because of enhanced OS in obesity, the risk of development of MS is even more elevated in overweight or obese subjects [26].

Recent studies have focused on whether OS and mitochondrial dysfunction are contributory factors for cellular and tissue damage in MS and type 2 diabetes. Both MS and type 2 diabetes are characterized by disturbances in fatty acid metabolism and accompanied by the accumulation of FFAs in non-adipose tissues. A large proportion of FFAs delivered by lipolysis in the mitochondria are attributed to the disorder in mitochondrial fuel metabolism, which is characterized by excessive β-oxidation, impaired switching to carbohydrate substrate, and decreased TCA cycle activity. This phenomenon results in incomplete oxidized products [71] that cause increased production of superoxide through the mitochondrial electron transport chain. Both humans and rodents with high dietary fat intake exhibit overproduction of superoxide in the mitochondria of skeletal muscle fibers [71]. The phenomenon further suggests mitochondrial overload as a direct mechanism by which excessive lipid supply leads to oxidative stress damage in MS and T2D. Increased oxidation of intracellular fatty acids also leads to increased mitochondrial NADH/NAD^+^ ratio and results in activation of the same mechanisms as hyperglycemia-induced ROS, including protein kinase C (PKC), advanced glycation end products, and NF-κB [72]. Hyperglycemia-induced ROS activates PKC, which in turns contributes to ROS production and OS by increasing the activity of NOX [73]. Other effects induced by PKC activation include inhibition of eNOS in endothelial cells [74], increased endothelial growth factor (VEGF) in vascular smooth muscle cells, and decreased NO production in smooth muscle cells [75]. Activation of PKC by hyperglycemia also induces TGF-β and NF-κB activation, which connect hyperglycemia-induced OS to inflammation [76].

Regarding the effects of advanced glycation end-products (AGEs), it has been reported that accumulation contributes to permanently altered cellular structure. Moreover, the activation of NAPDH oxidase, NF-κB, and pro-inflammatory pathways, as well as cytokine synthesis, have been speculated to be the primary mechanism by which AGEs promotes OS [77].

## 3. Oxidative Stress, Obesity and Type 2 Diabetes Mellitus

Type 2 diabetes mellitus is a condition characterized by elevated glucose levels in the blood which results from insulin resistance [78]. Obesity is a major driver of type 2 diabetes mellitus. The link between obesity and impaired serum glycemic levels indicates that progression towards diabetes occurs along a “continuum” which involves different cellular mechanisms including alterations of insulin signalling, changes in glucose transport, pancreatic β cell dysfunction, as well as enhanced OS and inflammation [79]. Hyperglycemia induces overproduction of ROS and DNA single-strand breaks. Moreover, the coexistence of obesity significantly contributes to the production of excess FR and ROS involved in diabetes and diabetic complications [78].

Despite several mechanisms, including the polyol pathway, PKC activation, accumulation of advanced glycation end products, and flux of hexosamine pathway [80], all being implicated in the tissue damage which occurs in diabetes mellitus, it would appear that all hyperglycemia-induced mechanisms are primarily activated by mitochondrial overproduction of ROS [81]. In cells with high intracellular glucose concentrations, higher amounts of glucose are metabolized and oxidized through the tricarboxylic acid cycle, thereby increasing the flux of NADH and flavin adenine dinucleotide (FADH2) into the mitochondrial electron transport chain, causing the accumulation of excess electrons to coenzyme Q, which eventually leads to superoxide generation [82]. Mitochondrial superoxide can amplify the damage by activating other superoxide production pathways. Studies have suggested that glucose can both directly stimulate ROS overproduction and also activate various enzymatic cascades in mitochondria, including activation of NADPH oxidase, uncoupling of NO synthases and stimulation of xanthine oxidase [83]. Therefore, glycated proteins may be the promoters of ROS formation [71]. Moreover, some evidence suggests that overproduction of ROS and decreased efficiency of antioxidant defenses start at a very early stage and eventually worsen over the course of the disease [81]. Recent data confirm that chronic elevation of intracellular ROS levels in adipocytes subsequent to mitochondrial dysfunction results in insulin resistance through attenuation of insulin signalling [84].

OS can also worsen β-cell dysfunction involved in the pathogenesis of type 2 diabetes, promoting glucotoxicity and lipotoxicity diabetes-related phenomena [72]. Furthermore, pancreatic β-cells exposed to hyperglycemia may produce ROS, which suppress glucose-induced insulin secretion [85]. Additionally, it has been reported that β-cells have relatively low expression of many antioxidant enzymes, making these cells susceptible to ROS-induced damage [86]. Confirming these results, other studies have shown that subclinical systemic inflammation in obese patients, as measured by elevated levels of CRP, IL-6, and TNF-α, predicts the development of type 2 diabetes [87,88] and contributes to a decrease of insulin sensitivity in peripheral tissues [89]. CRP is associated with insulin resistance while IL-6 may interfere with insulin signalling through induction of proteins that bind to the insulin receptor [87]. In addition, it has been reported that TNF-α is overexpressed in the adipose and muscle tissues of obese and insulin-resistant non-diabetic subjects, and overexpression is positively correlated with insulin resistance. Interestingly, serum TNF-α levels are also higher in type 2 diabetes patients [90].

IL-1 is thought to play an important role in the autoimmune destruction of pancreatic β-cells occurring in type 1 diabetes [91]. Nevertheless, IL-1 has also been implicated in the pathogenesis of type 2 diabetes as chronic inflammation contributes to the failure of β-cells to secrete sufficient amounts of insulin. Moreover, IL-1 synthesis has been noticed in pancreatic secretions obtained from subjects with type 2 diabetes. High plasma glucose levels further increase β-cell production and increased levels of IL-1, which in turn, in addition to TNF-α, stimulate the production of IL-6 [92]. Finally, the role of IL-1 in type 2 diabetes is further confirmed by Claus *et al.*, who demonstrated that IL-1 antagonism resulted in improved glycaemic control in subjects with type 2 diabetes [93].

In conclusion, in overweight or obese subjects, when adiposopathy, dysregulated function of adipose tissue [94], occurs, glucotoxicity and lipotoxicity act on pancreatic islet and liver and induce pancreatic β-cell dysfunction and liver insulin resistance, which are the decisive factors causing type 2 diabetes. Moreover, abnormal changes in the serum cytokine profile enhance the development and persistence of the diabetic state, which can be directly linked to obese status.

## 4. Oxidative Stress, Obesity and Cardiovascular Diseases

OS plays a crucial role in disorders related to obesity, such as dyslipidemia and hypertension, causing cardiovascular diseases (CVD).

### 4.1. Dyslipidemia

Dyslipidemia is defined as a condition of high blood cholesterol and TG levels that can increase the risk of CVD disease, stroke, and other health problems [95]. Obesity with dyslipidemia has been shown to promote the onset of CVD [96]. This link is strongly related to OS. Low levels of circulating high-density lipoprotein (HDL), enhanced clearance of HDL particles, increased post-prandial TG values, and elevated plasma very low density lipoprotein (VLDL) levels promote ROS generation in the endothelium [97]. In addition to a pro-inflammatory process, ROS can also directly damage lipids, proteins or DNA and modulate intracellular signalling pathways, such as mitogen activated protein kinases and redox sensitive transcription factors, causing changes in protein/lipid expression and, therefore, irreversible oxidative damage [97]. Due to ROS-mediated changes in lipid expression, further oxidation-derived products, including oxidative low-density lipoprotein (Ox-LDL), can play a further critical role in CVD. Ox-LDL, particles derived from circulating LDL that may have peroxides or their degradation products generated within the LDL molecule or elsewhere in the body [98], induces adipocyte proliferation either directly or indirectly by increasing the infiltration of monocytes/macrophages [99], by inducing the expression of lipoprotein lipase (LPL), and by inducing the accumulation of FA in adipocytes [100]. Additionally, Ox-LDL alters the production of adipokines which can lead to further OS. For example, Ox-LDL decreases the release of adiponectin, which inhibits ROS synthesis [101]. Increased Ox-LDL in obese patients with dyslipidemia may be due to loss of antioxidant capacity caused by low serum activity of the antioxidant enzyme (SOD) [12] or low HDL-associated paraoxonase-1 (PON-1), HDL attached extracellular esterase which contributes to the anti-atherogenic, anti-oxidant and anti-inflammatory properties of HDL [102]. Moreover, an increase in Ox-LDL could also be due to increased oxidant capacity, for example, by elevated expression of NOX2, which, in turn, induces further decreased production of adiponectin, increased pro-inflammatory cytokines levels, and generation of ROS in vascular and immune cells circulating in blood vessels. Furthermore, NOX-derived ROS interact with and stimulate other enzymatic sources of oxygen/nitrogen reactive intermediates, and amplify the initial response to insults mediated by FR [103].

In conclusion, although several mechanisms linking obesity and dyslipidemia (increased TG, Ox-LDL, and VLDL levels in addition to lower levels of circulating HDL) to CVD have been postulated, OS remains a major candidate implicated in vascular complication.

### 4.2. Hypertension

Human studies seem to support a role of OS in the development of hypertension, especially in obesity [78]. NO, released by the endothelium, causes vascular relaxation [104]. An imbalance in superoxide and NO production may account for reduced vasodilation, which can favour the development of hypertension. NO half-life is only a few seconds as it is rapidly degraded by the oxygen-derived free radical superoxide anion, released by eNOS, acting as a vasoconstrictor. As a result, eNOS may become a peroxynitrite generator, leading to a marked increase in OS with pleiotropic effects on vascular function by oxidation of cellular proteins and lipids [104]. The relationship of the degree of OS-induced alterations with blood pressure (BP) values was evaluated by Redon *et al.* [105]. In this study, in a group of untreated hypertensive subjects, oxidative status, antioxidant activities, and ROS byproducts in whole blood and mononuclear peripherals cells were measured in relation to BP values. They found increased OS and a reduction in the activity of antioxidant mechanisms independently of BP values [105]. Additionally, studies, using non-specific markers of oxidative damage, have observed a reduction in SOD and GPx activity inversely correlated with blood pressure in newly diagnosed and untreated hypertensive subjects, compared to healthy subjects [106]. Higher production of H_2_O_2_ has also been observed in treated and untreated hypertensive subjects compared to normotensive subjects, with a significant correlation between H_2_O_2_ levels and systolic blood pressure [107]. Moreover, both malignant and non-malignant hypertensive subjects had higher lipid hydroperoxide production [108].

Probably, the renin-angiotensin-aldosterone system (RAAS), including the sympathetic nervous system (SNS) stimulation of renin release, is also involved. Firstly, RAAS metabolites, such as angiotensin (Ang)-II and aldosterone, are potent vasoconstrictors contributing to hypertension. Growing evidence indicates that NADPH-driven generation of ROS and activation of reduction-oxidation (redox)-dependent signalling cascades are centrally involved in the role of Ang II-induced hypertension. Ang II, interacting with specific receptor Ang-II type 1 (AT1r), stimulates nonphagocytic NADPH oxidase, causing the accumulation of superoxide, H_2_O_2_, and peroxynitrite. Additionally, both sodium retention and activation of SNS, mediated by hyperinsulinemia, might enhance activation of RAAS and maintain hypertension [109]. Experimental models have reported that hypertension was correlated with elevated cerebral OS-activity and higher brain ROS levels [110,111]. Moreover, arterial blood pressure and SNS function are reduced by infusing antioxidants [111]. These data suggest that SNS excitation by OS in the brain could play an important role in the pathogenesis of obesity-associated hypertension. Finally, adipokines (leptin, adiponectin, ghrelin) as well as pro-inflammatory cytokines (TNF-α, IL-6 and IL-1), and neuropeptides (α-melanocyte-stimulating hormone and neuropeptide Y) have also been reported as further links between adipose tissue, OS and hypertension [112].

## 5. Oxidative Stress, Obesity and Liver Diseases

Nonalcoholic fatty liver disease (NAFLD) includes a broad spectrum of abnormalities (inflammation, fibrosis and cirrhosis), ranging from accumulation of fat (steatosis) to non-alcoholic steatohepatitis (NASH) [113]. NAFLD is part of MS, particularly in obesity, hyperlipidemia, and diabetes. The pathogenesis of NAFLD is not a simple mechanism and the theory of the “two-hit model” is the most widespread: The first hit is insulin resistant promoting hepatic fat accumulation, and the second hit, through a large number of adipokines (leptin, adiponectin, resistin), is sustained by FFAs that induce ROS injury [114]. The main element of NAFLD is the accumulation of TG as fat droplets within the cytoplasm of hepatocytes, which is a prerequisite for subsequent events of NASH. Increased delivery of both FFA and TG to the liver, decreased liver utilization of FFA, diminished export of TG from the liver and impaired β-oxidation of FFA within hepatocytes cause TG accumulation within the cytoplasm of hepatocytes [115,116]. Accumulation of lipids in the hepatocyte impairs oxidative capacity of mitochondria, increasing the reduced state of electron transport chain complexes, stimulating peroxisomal and microsomal pathways of fat oxidation. Additionally, mitochondrial dysfunction can directly lead to the production of ROS. If electron flow is interrupted at any point in the respiratory chain, the preceding respiratory intermediates can transfer electrons to molecular oxygen to produce superoxide anions and H_2_O_2_ [8]. Therefore, derived ROS can activate the Fas ligand/ Fas system, and progress to lead structural proteins of the Fas death zone to raise the downstream caspase family members to form the protease procascade reaction, resulting in cellular disorganization. The consequent increased generation of ROS and reactive aldehydic derivatives also promotes OS and cell death, via ATP, NAD, and glutathione depletion, and DNA, lipid, and protein damage [117]. Several other intracellular (e.g., mitochondrial dysfunction and endoplasmic reticulum (ER) stress) and extracellular (e.g., iron accumulation and inflammation by gut flora) factors (“triggers”) have been implicated in liver OS. ER allows synthesis and release of membrane proteins. For this function, high concentrations of intra-ER calcium are needed. Accumulation of FFAs, unesterified cholesterol, diacylglyceride and phospholipids induce a decrease of intra-ER calcium and an increase of “ER stress”, promoting apoptosis and hepatic stellate or Kupffer cells recruitment [118]. High serum FFAs levels also activate ketogenesis, mitochondrial, peroxisomal and microsomal FA oxidation, promoting the release of ROS which contribute to apoptosis and nuclear and mitochondrial DNA damage in NASH [118]. Regarding the role of iron overload in obese patients with NASH, it has been demonstrated that iron, via the Fenton reaction, plays a role in catalysing the production of ROS [119]. Additionally, over-expression of proteins binding iron in ER leads to local adipose tissue iron overload, inducing preconditions for adverse effects mediated by redox-active metal. Iron is capable of promoting OS, ER stress, inflammation as well as endocrine dysfunction. Therefore, iron-mediated mechanisms of toxicity may influence obesity pathogenesis and aggravate obesity related complications, including NASH [120]. Gut microbiota plays a critical role in NAFLD and NASH also in obese patients [118]. Recently, it has been demonstrated that gut microbiota of obese patients presents alterations in microbial composition. Through disrupted intercellular tight junctions and/or other pro-inflammatory bacterial products, it can favour intestinal inflammation, permeability and OS. This condition is sustained by increased pro-oxidant species (toll like receptors (TLRs), TNF-α) and decreased functionality of anti-oxidant mechanisms. All these pathways are involved in increased ROS production [121]. Increased production of ROS in the presence of excess FFAs has also been validated in animal models of NASH [8]. Human livers with NASH have increased levels of FFAs byproducts of lipid peroxidation, providing further evidence of an increase in OS in this condition [122]. Moreover, ROS and products of lipid peroxidation can lead to fibrosis by activating hepatic stellate cells, which synthesize collagen and perpetuate the inflammatory response, causing fibrogenic response [117]. This condition can be aggravated by low-grade chronic inflammatory in obese patients. Obesity-related cytokines, such as IL-6, TNF-α, as well as adiponectin, visfatin, and leptin [123] play important roles in the development of NAFLD, causing ROS-mediated hepatocellular injury. In particular, high serum levels of TNF-α and low levels of adiponectin are associated with major degrees of liver damage [124]. Studies have also demonstrated that hepatic steatosis leads to increased NF-κB. The latter induces further production of local and systemic inflammatory mediators (such as TGF-β, Fas ligand, TNF-α, leptin, adiponectin, IL-6, IL-1b, IL-8) involved in different lesions of NASH such as activation of Kupffer cells, macrophages, apoptosis, inflammation [125], and fibrosis 1 [126]. Finally, a liver with excess fat is more vulnerable to stressors, due to decreased antioxidant mechanisms [127], favouring OS-related obesity.

## 6. Oxidative Stress, Obesity and Cancer Susceptibility

An association between obesity and cancer has been reported across populations worldwide. A meta-analysis has shown that increased BMI was associated with a higher risk of both common and less common cancers [128]. In particular, in men, significant positive associations were also noted with rectal and prostate cancers. In women, positive associations were found with endometrial, gastrointestinal and post-menopausal breast cancers [128].

Several postulations have been made regarding the underlying pathophysiological mechanisms of cancer susceptibility in obese patients. These pathways have generally involved mechanisms related to genetic factors, insulin/IGF-I signalling axis, chronic low grade inflammation, adipokines secretion, and gut microbiota [129].

Genetic factors determine variation in BMI [130], associated with risk of many specific types of cancer [131] including gastrointestinal tumours, breast, prostate and thyroid cancers [131]. However, although genome-wide association studies have described altered “macrophage enriched metabolic network genes” [131,132] which are predisposing to cancer, other studies did not confirm these findings [133].

In response to hypothalamic signals, the liver releases IGF; specifically, human liver produces multiple isoforms of IGF and IGF-I is the most highly abundant isoform in circulation and is transported in the blood via IGF binding proteins (IGFBPs). Soluble IGF (IGFs) binds IGF receptors (IGFRs) and the insulin receptor (IR), expressed mainly in the gastrointestinal tract [134]. Here, IGF-I influences nutrient uptake through endocrine and neural pathways, and, stimulating cell division and inhibiting apoptosis, promotes cell proliferation [135]. Therefore, when the cellular activity of IGF-I is up-regulated, the risk of tumorigenesis and metastasis is strongly increased [136].

Obese patients exhibit lower levels of antioxidant enzymes and increased concentrations of OS byproducts. Obesity increases the size and activity of adipocytes, leading to release of inflammatory molecules (e.g., IL-17, IL-22, TNF-α, IL-6, and monocyte chemoattractant protein 1 (MCP-1), tissue necrosis and subsequent accumulation of activated macrophages. This pro-inflammatory status promotes, in turn, the development of insulin resistance [137], down-regulation of anti-inflammatory factors (IL-10, IL-4, TGF-α, T-regs) [138], increase of ROS, inducing oxidative modification of critical macromolecules [139], and, finally, carcinogenesis [135]. Due to this, chronic inflammation is more pronounced in visceral than in subcutaneous fat compartments, and explains a major incidence of neoplasia in obese patients.

Adipose tissue synthesizes and secretes several adipokines, which can alter metabolic cellular function [140]. Altered leptin, and adiponectin levels have been associated with cancer development. Leptin, produced in response to lipopolysaccharides, insulin, sex hormones, and pro-inflammatory mediators (IL-1b, IL-6, TNF-α), binds to transmembrane receptors on stomach, colon, estrogen-dependent breast cancer [141], androgen-insensitive prostate [142], and thyroid cells [143], resulting in angiogenesis, cellular proliferation, migration, and invasion of tumour cells, and inhibits apoptosis [144]. The actions of leptin on cell functions are balanced by adiponectin [145], having antiproliferative and antiangiogenic effects [146]. In fact, hypoadiponectinaemia is known as risk factor for tumorigenesis [147].

Gut microbiota impacts obesity through its capacity to increase caloric salvage of indigestible dietary polysaccharides, regulate intestinal genes promoting fat storage [148], and induce gastro-intestinal inflammation [149], which could contribute to obesity-associated gastro-intestinal carcinogenesis. The composition of the intestinal microbiota could be involved in the development of small and large intestine, esophageal, gastric, and even pancreatic neoplasms [148,149].

In conclusion, the prevalence of obesity has significantly increased the risk of developing cancer. To date, although the association between obesity and tumours is not always congruent, there is a significant amount of data showing that obesity, especially visceral abdominal obesity, is an important risk factor for tumour development.

## 7. Conclusions

Obesity, a social problem worldwide, characterized by an increase in body weight that results in excessive fat accumulation, has been recognized as a major underlying factor of the pathogenesis of several diseases (e.g., metabolic syndrome, diabetes mellitus, cardiovascular and liver diseases, and cancer) [2], all-cause mortality, and a reduced life expectancy [150]. All these pathological conditions are associated to OS. Because of “inflamed fat” [151,152], obesity is also correlated to similar inflammatory conditions. Therefore, it has been hypothesized that inflammation of adipose tissue, in obese patients, plays a critical role in the pathogenesis of obesity-related complications. The association of obesity with obesity-related inflammatory diseases can be explained by the following mechanisms: (i) In obese animals or humans, adipose tissue is characterized by increased local and systemic production of pro-inflammatory adipocytokines [153], which induce the production of ROS; and (ii) Increased OS leads to important changes in adipose tissue that promotes a systemic low-grade inflammatory response with adverse effects throughout the body [27]. Although recent important contributions have been made in this field, future studies should address the potential role of OS in obesity to regulate the onset and progression of autoimmune and/or inflammatory conditions. Furthermore, in light of data in the literature, it should be noted that OS in obesity requires higher consideration, especially in children. Infants may exhibit peculiar susceptibilities to the effects of OS as they are undergoing rapid tissue growth and development. Additionally, although OS occurs early in life [152,154], it predisposes the younger population, with longer life expectancy, to favour diseases with long latency periods. Finally, an understanding of the molecular mechanisms of obesity-associated conditions would be useful to the development of new therapies, and for preventing several diseases.
